# Bridging the Gap Between Second Language Acquisition Research and Memory Science: The Case of Foreign Language Attrition

**DOI:** 10.3389/fnhum.2019.00397

**Published:** 2019-11-13

**Authors:** Anne Mickan, James M. McQueen, Kristin Lemhöfer

**Affiliations:** ^1^Donders Institute for Brain, Cognition and Behaviour, Radboud University, Nijmegen, Netherlands; ^2^International Max Planck Research School for Language Sciences, Max Planck Institute for Psycholinguistics, Nijmegen, Netherlands; ^3^Max Planck Institute for Psycholinguistics, Nijmegen, Netherlands

**Keywords:** foreign language attrition, forgetting, retrieval-induced forgetting, interference, competition, second language acquisition, memory

## Abstract

The field of second language acquisition (SLA) is by nature of its subject a highly interdisciplinary area of research. Learning a (foreign) language, for example, involves encoding new words, consolidating and committing them to long-term memory, and later retrieving them. All of these processes have direct parallels in the domain of human memory and have been thoroughly studied by researchers in that field. Yet, despite these clear links, the two fields have largely developed in parallel and in isolation from one another. The present article aims to promote more cross-talk between SLA and memory science. We focus on foreign language (FL) attrition as an example of a research topic in SLA where the parallels with memory science are especially apparent. We discuss evidence that suggests that competition between languages is one of the mechanisms of FL attrition, paralleling the interference process thought to underlie forgetting in other domains of human memory. Backed up by concrete suggestions, we advocate the use of paradigms from the memory literature to study these interference effects in the language domain. In doing so, we hope to facilitate future cross-talk between the two fields and to further our understanding of FL attrition as a memory phenomenon.

## Introduction

In 2016, more than 60% of adult European citizens were able to speak *at least one* foreign language (FL; European Commission—Eurostat, 2016). With multilingualism on the rise, learning foreign languages (FLs) is so common these days, it is often taken for granted. Yet, regardless of how ordinary it might seem, mastering a new language is and always will be an immensely complex task. Being able to formulate sentences in any language requires knowledge of its words and grammatical structures, all of which have to first be encoded, and then consolidated and integrated into long-term memory. All of these processes are common to other types of learning as well and are ultimately underpinned by the same fundamental memory processes. Surprisingly, despite the obvious overlap between second language processing and memory function, the empirical investigations of the two have often gone on in parallel; and so for a long time the fields of second language acquisition (SLA) and memory science developed in isolation from each other^1^. This is also true for the study of FL attrition, which investigates the phenomenon of forgetting a previously mastered FL. While it is not new to apply memory theories of forgetting and their corresponding paradigms to (FL) attrition, they have, as we shall argue, not been used to their full potential. Taking FL attrition as an example, we will posit that both SLA and human memory could benefit from more cross-talk. In doing so, we encourage future research exploiting parallels between the two fields.

### Previous Research on Foreign Language (FL) Attrition

In the past 50 years, researchers have gone to great lengths to document language forgetting. Most of this research has been directed towards first language (L1) attrition in migrants (for reviews on L1 attrition, see Köpke and Schmid, 2004; Schmid, 2016). Much less work has been dedicated to FL attrition, the forgetting of a language learned later in life (for an overview, see Schmid and Mehotcheva, 2012). The present article will focus on this latter type of attrition, partly because it is a less well studied and hence less well-understood type of attrition; but also because, as will become apparent later on, the approach we are advocating in this article is most directly applicable to the FL attrition context.

FL forgetting often first manifests in a decrease in fluency and lexical diversity in the FL (Bardovi-Harlig and Stringer, 2010). Consequently, the majority of studies on FL attrition have focused on the lexicon (i.e., vocabulary), leaving (morpho-) syntax and phonology aside (but see for example, Berman and Olshtain, 1983; Hedgcock, 1991; Dugas, 1999; Tomiyama, 2008). Given this bias in the existing literature, most examples given below will pertain specifically to *lexical* FL attrition, though we will speculate about the applicability of our proposed approach to other types of attrition as well (see “Going Beyond Foreign Language Vocabulary Attrition” section).

Based on the existing FL attrition literature, forgetting seems to set in very quickly after one stops using a FL; yet it then gradually levels off, with the most basic vocabulary apparently preserved in so-called “permastore” (Bahrick, 1984). It furthermore appears that productive skills deteriorate faster than receptive skills (e.g., Bahrick, 1984; De Groot and Keijzer, 2000), and that one tends to first lose the information learned last (or possibly what has been consolidated or practiced the least; i.e., the “regression hypothesis (RH)”; e.g., Cohen, 1986; Olshtain, 1989; Kuhberg, 1992; see “Discussion and Directions for Future Research” section for a more in-depth discussion of this hypothesis and its alternative formulations). Next to those commonalities, attrition differs heavily from person to person. Exactly how severe and fast the attrition process is depends on a variety of factors. Bahrick’s (1984) study on school-learned Spanish, for example, showed that those individuals with the highest Spanish proficiency before attrition onset were least affected by forgetting (see also Weltens, 1988; Murtagh, 2003; Mehotcheva, 2010). Age at attrition onset (Olshtain, 1989; Bardovi-Harlig and Stringer, 2010) as well as language usage and exposure patterns (e.g., Mehotcheva, 2010) are also believed to play a role in determining the course of FL attrition: the earlier one stops learning the FL and the less exposure one has to it afterward, the more likely one is to suffer from attrition in that language. Finally, one’s attitude towards the target language as well as motivation to maintain it is also believed to influence its attrition rate (e.g., Mehotcheva, 2010). Research on especially the latter two variables’ role in attrition, however, has often yielded contradictory results (see Schmid and Mehotcheva, 2012), thus calling for more research into the determinants of attrition.

Interestingly, in most cases FL attrition (just as in fact any type of attrition) appears to be temporary and reversible: while one may not consciously recall the words learned in French class in high school, studies have demonstrated that relearning seemingly lost FL vocabulary is much easier and faster than learning new FL vocabulary from scratch (e.g., Hansen et al., 2002; de Bot et al., 2004). Such relearning advantages indicate residual storage of the purportedly “forgotten” words and thus speak against complete loss of memory traces. Attrition, like any other kind of forgetting, is thus best understood as a performance problem, characterized by accessibility difficulties rather than actual loss (Sharwood Smith, 1989).

Relatedly, it should be noted that observed FL attrition rates depend heavily on how the FL knowledge is tested: while attriters may be unable to freely recall and produce a word, they might still be able to recognize the word in a lexical decision task or other recognition-based tests. In other words, as mentioned earlier, productive recall failure appears to precede recognition inability. In earlier studies on FL attrition, the focus was often on receptive vocabulary knowledge, as this was thought to give the clearest picture of a person’s existing FL knowledge, but those studies often reported very little to no attrition even after years of no exposure (e.g., Weltens et al., 1989; Grendel, 1993). These null results stand in stark contrast to studies reporting significant attrition in productive recall tasks already within the first year of disuse (e.g., Bahrick, 1984; Mehotcheva, 2010). This distinction needs to be kept in mind in interpreting differences in attrition rates between populations and studies.

### Forgetting Due to Competition and Interference: A Domain-General Perspective

The above studies have made important contributions to our understanding of language attrition. It remains unclear, however, what exactly causes language forgetting, and thus which cognitive mechanisms underlie it. Forgetting is by no means limited to language though, and is, in fact, a rather pervasive phenomenon: we forget where we park our car, or what that distant friend’s name was. Research on forgetting from a more domain-general perspective dates back to the 19th century and Ebbinghaus’ research on the ease of learning and relearning nonsense-syllable sequences (Ebbinghaus, 1885, 1913). Ebbinghaus discovered that memory loss was not linear over time, but logarithmic instead: most forgetting happens over the first minutes to hours and then gradually levels off; note that this resembles what Bahrick (1984) observed for the retention of school-learned Spanish. Ebbinghaus’ work inspired many theories about the possible mechanisms behind forgetting (for an overview, see Ecke, 2004; Anderson, 2015). The probably most influential of these is interference theory. Rather than assuming that forgetting is a by-product of time (see decay theory, Thorndike, 1914), interference theory attributes forgetting to interference from related, competing memories. Essentially, it relies on the fact that memories that share a common retrieval cue (e.g., your car being the shared cue for its location today vs. yesterday) compete with one another for selection upon presentation of that cue, thus hindering future retrieval.

One example of forgetting by competition is retrieval-induced forgetting (RIF; Anderson et al., 1994). In a typical RIF study, participants first study a number of category-exemplar pairs (e.g., FRUIT-apple, FRUIT-banana, FURNITURE-table). This phase is followed by selective retrieval practice of some exemplars from some of the categories (FRUIT-banana, but not FRUIT-apple or FURNITURE-table). Finally, recall is tested for all originally studied pairs. Of course, recall is best for the practiced pairs (FRUIT-banana), but interestingly, it is worse for unpracticed exemplars from practiced categories (FRUIT-apple) compared to recall for unpracticed exemplars from unpracticed categories (FURNITURE-table). The mere act of retrieving information can thus hamper access to information related to the practiced material. RIF is typically attributed to executive control processes in the form of inhibition applied to competitors during the retrieval practice phase (e.g., to apple), making these suppressed competitors harder to retrieve at final test (e.g., Anderson, 2003; Bäuml et al., 2005; Román et al., 2009; though see Williams and Zacks, 2001; Raaijmakers and Jakab, 2013; for alternative explanations). RIF effects have been demonstrated with a wide variety of stimulus materials. It thus appears to be a generalizable phenomenon (for a review, see Storm et al., 2015).

## Exploring Parallels Between Memory and SLA Research

Competition does not only exist between exemplars of a semantic category, but also between translation equivalents in different languages that share a common concept. When a speaker of English and Spanish wants to refer to a “table,” both Spanish “mesa” and English “table” will be activated and compete for selection. This between-language competition is well-known to affect (online) word production in bilinguals (e.g., Hermans et al., 1998; Colomé, 2001; for an overview, see Kroll et al., 2008). As in RIF, bilingual lexical access is often seen as a matter of executive control: it is assumed that in order to avoid unwanted language selection/production errors, speakers need to inhibit the non-target language during speaking. This, however, can, as an undesirable side effect, lead to later retrieval difficulties in the inhibited language (Green, 1998). In terms of competition for retrieval, translation equivalents (sharing the same concept) are thus similar to pairs of exemplars sharing one semantic category cue. Given this parallel, the question arises whether between-language competition is also a driving mechanism behind language forgetting, and thus whether the between-language competition observed during the short-term, online processing also has long-term ramifications.

### Between-Language Interference as a Mechanism Behind FL Attrition?

The idea that attrition is the result of complex interactions and competition between languages is not new. Sharwood Smith (1989) as well as Seliger and Vago (1991) already noticed how *L*1 *attrition* is influenced by the newly acquired FL (L2), for instance in the form of code switches to L2 while speaking in L1. This “cross-linguistic influence hypothesis” (Sharwood Smith, 1989) is also central to more recent approaches to attrition. Its ideas have been formally discussed, for example, within the context of Paradis’ Activation Threshold Hypothesis (ATH, see Köpke, 2002; Gürel, 2004; Paradis, 2004, 2007). The ATH assumes that all items in the linguistic system, such as words, are interconnected and influence one another. Each item has an activation threshold (AT). Retrieving a word requires that its activation exceed its AT. The AT is lowered after successful retrieval but is increased again either gradually through disuse or through top-down inhibition during access of other, competing words (as is also believed to be the case in RIF; Anderson, 2003). A latter mechanism is a form of cross-linguistic influence since competing items will often be translation equivalents in other languages. Heightened ATs then lead to retrieval difficulties because more activation is needed to pass them. Ultimately, ATs can be so high that a word can no longer be accessed, and hence is “forgotten” until accessed again (e.g., during re-learning). For Paradis (L1 lexical), attrition is thus the result of a lack of stimulation of certain words, combined with more recent and frequent access of competing for translation equivalents^2^.

Applying ideas and frameworks like the ATH to attrition forms part of an increasing effort towards studying the phenomenon from a psycholinguistic perspective (see also Schmid and Köpke, 2017). Surprisingly though, only a handful of psycholinguistically-inspired attrition articles explicitly connect their ideas to memory theories of forgetting. In an effort to encourage more such cross-talk, Ecke (2004) summarized theories of forgetting from the memory literature and stressed their theoretical relevance and explanatory value for attrition (see also Köpke, 2004). While discussing multiple theories of forgetting, he identifies interference processes as the “main contributor to attrition” (Ecke, 2004; p. 337). We aim to build on Ecke’s (and Paradis’) contribution, but go one crucial step further: next to using memory theories of forgetting as a framework within which to think about attrition, we argue that an important added value of these theories are the *experimental paradigms* that have been developed to test them. The application of these paradigms to the study of (at least FL lexical) attrition forms a crucial addition to the available evidence on FL attrition. *Via* snapshots of FL ability at different time points, existing observational studies document merely the result, but not the process of attrition itself. Observed links between, for example, language use (as typically measured in questionnaires) and attrition are then purely correlational and do not indicate causal relationships. Traditional attrition studies thus cannot be taken as proof that any factor discovered in this manner is a driving force in attrition. The memory approach to the investigation of forgetting is quite different from the traditional attrition approach: experimental paradigms (e.g., the RIF paradigm) aim at inducing and thus simulating forgetting in a tightly controlled setting. To do so, cognitive psychologists typically manipulate the presence or absence of a presumed cause of forgetting (e.g., interference) while keeping all other potentially relevant factors (e.g., amount of studied materials) constant. By determining the conditions that do and do not lead to forgetting, this procedure allows for *causal* inference.

This experimental approach can be applied to language attrition. Retrieval of words in one language should induce retrieval difficulties, and thus ultimately forgetting of translation equivalents in another language. A handful of studies have put this hypothesis to test, among those a recent study by Mickan et al. (submitted). Participants first learned a set of new L3 Spanish words. A day later, interference was introduced: participants were asked to retrieve half of these newly learned words in either L1 Dutch or L2 English; the other half were not interfered with. Finally, after a 20-min delay, all originally learned words were tested again in Spanish. In line with predictions based on interference theory, participants were worse at recalling interfered words compared to not-interfered words. That is, retrieving translation equivalents in other languages made them forget (some of) the recently learned Spanish words. This interference effect was also visible in naming speed for the words that were still successfully remembered: it took participants longer to retrieve interfered compared to not-interfered words. In reaction times, this effect persisted in a follow-up Spanish test a week later, thus showing that interference has true long-term effects and establishing it as a plausible mechanism behind FL attrition.

Similar observations had previously been made for L1 attrition, for which Levy et al. (2007) had shown that retrieval practice in L2 Spanish impaired subsequent recall for L1 translation equivalents. For FL attrition, there are two corroborating studies with similar results. Bailey and Newman (submitted) tested L1 English learners of L2 Welsh and showed that participants were slower to recall newly learned Welsh words when these words had intermittently been retrieved in L1. Likewise, Isurin and McDonald (2001) found that memory for newly learned L2 Russian words was worse if participants had in between learned the same words in L3 Hebrew as compared to when no extra learning had taken place. Note though that neither Bailey and Newman (submitted) nor Isurin and McDonald (2001) allowed for consolidation of the newly learned Welsh or Russian L2 words: finding interference in these cases is less surprising given that the newly learned material had no chance to consolidate. Moreover, neither of these studies had a delay between “interference” and final recall, thus not providing evidence for long-term interference effects (for which a delay of at least 20 min is called for, following standard memory procedures; Anderson et al., 1994). Both these studies are thus somewhat more removed from real-world attrition scenarios and less convincingly link interference to long-term forgetting than Mickan et al. (submitted).

Delays of up to 1 week, as tested in Mickan et al. (submitted), might seem minuscule compared to the time delays of multiple months or even years that are typically reported in observational attrition studies. In experimental terms, however, it is quite remarkable for effects to persist for an entire week. While looking at longer time delays would be theoretically interesting for future studies, doing so only makes sense if: (1) it can be guaranteed that the participants are not re-exposed to the target language within that time; and (2) only if additional interference can be reliably quantified. If these two conditions are not met, the experimenter would no longer have the experimental control that makes the simulation approach so useful. What is more, it would be difficult to interpret the outcome of a longer time delay: additional interference through the intermittent use of other languages would happen equally often for items in the interference and no interference conditions, and so would wash the interference effect out. The experimentally induced interference effect might thus disappear with time, however, *not* necessarily because it is not long-lasting, but instead because of additional interference, the very mechanism that caused the effect in the first place. There is thus a logical limit to the length of the delays one can sensibly look at while maintaining experimental and explanatory control; and 1 week is arguably already stretching this limit.

Overall, while there is clearly a need for more studies to establish interference-based forgetting as a robust phenomenon in the language domain, the above-cited studies illustrate that using paradigms from the memory literature complements more traditional approaches to attrition, allows for causal rather than just correlational inferences and thereby advances our understanding of why we forget languages.

## Discussion and Directions for Future Research

We have argued that the use of memory paradigms for the study of attrition has a number of advantages over more traditional approaches. Simulating rather than observing attrition in real life makes it possible to control, isolate and manipulate possible determinants of the attrition process and assess their effect on retention rates. Most obviously, this includes manipulations of the interference phase as the phase during which attrition is (presumably) taking place. As a secondary question, Mickan et al. (submitted), for example, asked whether the *type* of interference matters: manipulating interference language between experimental groups, they found that another FL (L2 English) interfered more with L3 Spanish than the participants’ dominant L1. Along similar lines, one might ask what the role of typological proximity or language distance is in driving attrition. Do two related languages interfere more with one another than two distant ones? For vocabulary, this question translates to a comparison of cognates (i.e., words that share meaning and form across languages) and non-cognates. Cognates have often, not surprisingly, been reported to be less affected by attrition than non-cognates (e.g., Weltens, 1988). This assumption is in line with interference theory: since cognates share form and meaning, there is no need to suppress the translation equivalent when retrieving a cognate in the target FL. One might even expect a boost for identical cognates given the form overlap. Often, however, words in typologically similar languages overlap only partially (e.g., “table” in English and “Tafel” in Dutch). It is unclear whether such non-identical cognates interfere more or less with each other than two non-cognate translation equivalents. This could be addressed in an experiment that compares interference effects for the two types of nouns. Given that, by definition, two typologically similar languages will have relatively more (non-identical) cognates than two distant languages (Chiswick and Miller, 2005), stronger (or weaker) interference effects between non-identical cognates as compared to non-cognates would be evidence in favor of (or against) the hypothesis that two typologically close languages interfere more with one another than more distant languages.

Likewise, it would be interesting to test whether active use of other languages is necessary to induce forgetting, or whether mere passive exposure to other languages is enough. Evidence from memory studies seems to suggest that active retrieval and response generation (though not necessarily successful retrieval, Hellerstedt and Johansson, 2016) is necessary to induce RIF; passive exposure or even reading out loud of exemplar-pairs does not induce forgetting of related items (Anderson et al., 2000; Bäuml, 2002). It remains to be seen whether these findings generalize to the attrition context.

Memory paradigms might also help answer long-standing open questions regarding FL attrition. The RH is a case in point: in its original formulation, RH posits that we tend to forget first the information (e.g., words) we learned last (Jakobson, 1941). However, the order of acquisition itself might not actually matter as much as the degree of learning of a given word (i.e., “best learned = last forgotten”; Hedgcock, 1991). In the real world, these two theories are almost impossible to tease apart: with more time for rehearsal and repetition, remotely learned words will be better encoded than recently learned words. To worsen matters further, the first words one learns in a new language tend to be the most frequent; later learned words or structures instead are usually less frequent, harder to learn, and possibly more vulnerable to forgetting because of their difficulty rather than order of acquisition. A lab study could disentangle these options by manipulating the acquisition order during the initial learning phase while keeping the amount of exposure (and thus the degree of learning) for each word—as well as subsequent interference—equal.

Another option would be to compare receptive and productive recall abilities. As mentioned in “Previous Research on Foreign Language Attrition” sections, it is generally assumed that productive loss precedes receptive loss. Support for this claim often comes from comparisons across studies, that is between different groups of participants and even different language combinations (though see Bahrick, 1984; De Groot and Keijzer, 2000; for exceptions). The above-reported studies (all testing productive recall with the exception of Bailey and Newman, submitted) could easily be adjusted to test both productive *and* receptive recall at final test (e.g., *via* a lexical decision test in addition to a picture-naming test) and hence could be used to *directly* compare the two. Finding that words that are already “forgotten” in productive tasks are still available to the participant in receptive tasks would be much more convincing proof of the claim that receptive knowledge outlasts productive recall ability than the cross-experiment comparisons that have often been the basis for this claim in the past.

Yet another possible future research avenue concerns manipulations of the level of FL proficiency reached prior to attrition onset. Higher ultimate attainment, as it is often called, has consistently been linked to better retention (see Schmid and Mehotcheva, 2012). Yet, it is unclear whether this means that highly proficient FL learners really attrite less, or whether they, in fact, attrite equally much in absolute terms, but are left with a larger vocabulary because they had a bigger lexicon to begin with (Bahrick, 1984, supports the latter). Most studies to date cannot disentangle these two options because they often do not have the necessary baseline measurement (with the exception of longitudinal studies, yet those tend to be underpowered). A simulation study might again help to disentangle the two: one could test two groups of participants, one as described above, and one with an extended initial learning session (possibly spread over multiple days and/or with an increase in the number of words to be learned) to simulate a higher FL proficiency level, while keeping the amount of interference constant. It should get harder to simulate attrition in the lab as FL proficiency increases if higher ultimate attainment really leads to less attrition. Crucially though, comparing the low and high attainment groups will reveal whether forgetting rates are comparable or actually different across different levels of ultimate FL attainment.

Finally, lab studies could be used to investigate individual differences in attrition. With a large enough pool of participants, there is bound to be variability in forgetting rates, even in an otherwise tightly controlled lab study. It would be interesting to test whether factors known to modulate RIF and interference resolution in bilingual processing also play a role in determining the rate of interference-induced FL attrition. An interesting case in point is executive/cognitive control ability, which has been found to be implicated both in bilingual processing (e.g., Linck et al., 2008) and RIF (e.g., Mall and Morey, 2013; though not always to same extent or even in the same manner, see Aslan and Bäuml, 2011). Traditional attrition studies have, to our knowledge, paid little attention to cognitive control ability. Should it turn out to be a reliable predictor of forgetting rates in the lab, it would merit investigation in large scale studies with real attriters; and might explain some of the residual variances that remain unexplained by the otherwise mostly socio-linguistic variables assessed in previous studies.

As these examples show, the possibilities using variations of memory paradigms are manifold. Of course, as with any approach, there are also downsides: using tightly controlled experiments clearly comes at the cost of ecological validity. Attrition is a complex, multi-faceted phenomenon, which is simplified in the above experiments. There are undoubtedly questions that our approach will not be able to answer (e.g., motivational/attitudinal aspects). Before looking at large complexities, however, one needs to understand the basic mechanisms. The power of lab studies lies in making exactly that possible: by simplifying matters and isolating individual factors, they can substantially contribute to the development of a cognitive theory of attrition, uncontaminated by the noise that is inevitable in observational studies. Of course, the mechanisms unraveled in the lab will then need to be verified by large-scale longitudinal studies with real attriters. Such studies need to keep detailed records of all possible determiners of attrition, like their participants’ language usage patterns, for example, *via* questionnaires or more formal tasks at regular and *short* intervals (monthly at least). While it will remain challenging to recruit a large enough, homogenous sample of “natural” attriters, we would like to highlight the possibility of online testing nowadays. There are many types of measures that can be taken online (both receptive and productive tasks are possible), and one can much more easily reach a large number of people, which would ultimately allow for firmer conclusions if patterns emerge reliably. Hence, we do not propose that experimental studies should replace traditional ones. Instead, we see the two approaches as complementary and believe there should be a healthy balance between them.

### Going Beyond Foreign Language Vocabulary Attrition

#### Syntax

The present article focuses on FL lexical attrition. Yet, speaking a language requires much more than just mastery of its words. Similarly, attrition is by no means limited to the forgetting of vocabulary; grammatical structures have, for instance, also been found to attrite (e.g., Hansen, 1999; Tomiyama, 2008). It will be crucial to extend the current line of research to cover these other types of attrition as well. As a first step, one could look at grammatical gender, for which negative transfer and interference effects are well documented in online processing (e.g., Lemhöfer et al., 2008). One might ask whether retrieving L1 gender for a set of nouns (e.g., “der Mond,” masculine in German) interferes with and makes people forget just recently learned, but incompatible FL gender assignments (e.g., “la luna,” female in Spanish).

For more rule-governed aspects of grammar, the design might need some adjustments: the learning phase, for example, will most likely need to be longer, and include tasks other than just picture naming for participants to learn the rule. Moreover, the control (i.e., no-interference) condition will need to be carefully chosen: if the syntactic property is not item-specific (unlike grammatical gender), one would need to find a syntactic rule that is comparable in complexity, yet not in conflict with and thus not prone to interference from L1. This might prove challenging for some aspects of grammar. In such cases, one might need to resort to between-subject designs and compare a group that learns a conflicting rule with a group that learns the same rule but in a language which implements this rule similarly to their L1. Though somewhat more challenging, we think that extending our approach to syntax would be a very interesting line of research.

#### First Language Attrition

While almost all the above studies are concerned with FL attrition, Levy et al.’s (2007) research suggests that L1 attrition can also be experimentally induced. The design of a study on L1 attrition differs slightly from the FL attrition studies reported above though: there is no need for an initial learning phase in L1; one instead would start with a baseline L1 picture naming test. This baseline speed and accuracy measurement would then be followed by an interference phase that could consist of learning of some of the same words in a new FL. Finally, retrieval speed and accuracy would be measured again for all words in L1. While such a study is perfectly conceivable, it remains unclear how easily deeply engrained L1 knowledge can be interfered with. Even though Levy et al. (2007) observed worse L1 English recall rates after L2 Spanish retrieval practice, Runnqvist and Costa (2012) were later unable to replicate this finding. What is more, Levy et al. (2007) used a rather indirect measure of recall ability (rhyme-generation rather than picture naming), which possibly underestimated L1 productive knowledge. For successful L1 attrition induction, as measured in a final picture naming task, the interference phase might need to be longer, or spaced out over multiple days. Even if L1 attrition proves to be inducible in the lab though, it should be mentioned that the L1 words under investigation will have been learned in the wild and *not* under controlled circumstances. Hence, there will be limitations to the types of simulations one can run; some of the questions addressed in “Going Beyond Foreign Language Vocabulary Attrition” section will be impossible to implement for L1 attrition (e.g., disentangling the effects of order of acquisition vs. degree of learning of L1 words on L1 attrition rates).

### Bridging Between Memory and SLA—a Two-Way Street

Finally, we would also like to emphasize that the benefit of applying psychological theories of forgetting to the language attrition context is not a one-way street. Traditional memory paradigms often make use of artificial learning materials that are hardly representative of what people learn outside the laboratory (e.g., word lists, association pairs, or visual patterns). FL learning and forgetting offers a more realistic scenario to memory researchers to test their theories on. This advantage should not be overlooked given that the FL scenario is arguably as close to real-life as memory studies on the topic can get, while maintaining tight experimental control.

### Concluding Remarks

We hope to have shown that the approach of using experimental paradigms from human memory research to study FL attrition is a promising avenue for future research. It provides a fresh look at FL attrition that allows for very different types of inferences than those supported by traditional observational studies. We believe that a sound mixture of both approaches is needed if we are to understand what it means to forget a FL. The field of FL attrition is only one example out of many in SLA research for which such interdisciplinary cross-talk is relevant (e.g., effects of testing, spacing and later consolidation for FL vocabulary learning). We hope to have contributed our share to a productive bridging between SLA and memory science.

## Author Contributions

AM wrote and edited the manuscript. KL and JM provided feedback and edited the manuscript. All authors read and approved the submitted version.

## Conflict of Interest

The authors declare that the research was conducted in the absence of any commercial or financial relationships that could be construed as a potential conflict of interest.

## Footnotes

^1^We use the terms “second” and “foreign” language interchangeably in this article. Both refer to any language other than one’s mother tongue (L1); that is any language learned later in life, be it a second (L2), third (L3) or even fourth foreign language. In using both terms we stick to common terminology in both “*second* language acquisition” as well as “*foreign* language attrition” research.

^2^Paradis (2004) also distinguishes between declarative (i.e., explicit) and procedural (i.e., implicit) memory. Declarative memory encompasses knowledge of facts and events and is accessed consciously; procedural memory refers to memory for skills and is largely unconscious (Squire, 1992). Paradis (2004) assumes the language faculty is subserved by both these types of memory as well, with any language’s vocabulary and the majority of FL grammar being instances of declarative memory, and L1 grammar mostly procedural (also see Ullman, 2001, 2004). For attrition, Paradis further speculates that declarative memory, and hence L1/FL vocabulary and FL grammar, will be more prone to interference and forgetting than aspects of procedural memory. The present article is on *lexical FL* attrition and thus concerns the *declarative* memory system only.
